# Lung cancer screening with volume computed tomography is cost-effective in Greece

**DOI:** 10.1371/journal.pone.0316351

**Published:** 2025-03-05

**Authors:** Xuanqi Pan, Katerina Togka, Hilde ten Berge, Lisa de Jong, Harry Groen, Maarten J. Postma, Eleftherios Zervas, Ioannis Gkiozos, Christoforos Foroulis, Kyriaki Tavernaraki, Sofia Lampaki, Georgia Kourlaba, Antonios Moraris, Sofia Agelaki, Konstantinos Syrigos

**Affiliations:** 1 Institute for Diagnostic Accuracy, Groningen, The Netherlands; 2 Unit of Global Health, Faculty of Medical Sciences, University of Groningen, Groningen, The Netherlands; 3 Department of Pulmonary Diseases, University of Groningen, University Medical Center Groningen, Groningen, The Netherlands; 4 Department of Economics, Econometrics & Finance, Faculty of Economics & Business, University of Groningen, Groningen, The Netherlands; 5 7th Pneumonology Department, Sotiria Thoracic Diseases Hospital of Athens, Athens, Greece; 6 Third Department of Internal Medicine, Sotiria General Hospital for Chest Diseases, National and Kapodistrian University of Athens, Athens, Greece; 7 Department of Cardiothoracic Surgery, Aristotle University of Thessaloniki, AHEPA University General Hospital, Thessaloniki, Greece; 8 Imaging and Interventional Radiology, Sotiria General and Chest Diseases Hospital, Athens, Greece; 9 Department of Pneumonology, “Papanikolaou” Hospital, Thessaloniki, Greece; 10 Department of Nursing, Faculty of Health Sciences, University of Peloponnese, Tripoli, Greece; 11 External Affairs Department, AstraZeneca Greece, Athens, Greece; 12 Department of Medical Oncology, University Hospital of Heraklion, Heraklion, Greece; Affiliated Hospital of Nantong University, CHINA

## Abstract

**Objective:**

This study aimed to assess the cost-effectiveness of lung cancer screening (LCS) employing volume-based low-dose computed tomography (LDCT) in contrast to the absence of screening, targeting an asymptomatic high-risk population in Greece, leveraging the outcomes derived from the NELSON study, the largest European randomized control trial dedicated to LCS.

**Methods:**

A validated model incorporating a decision tree and an integrated state-transition Markov model was used to simulate the identification, diagnosis, and treatments for a population at high risk of developing lung cancer, from a healthcare payer perspective. Screen-detected lung cancers, costs, life years (LYs), quality-adjusted life years (QALYs), and the incremental cost-effectiveness ratio (ICER) were predicted. Sensitivity and scenario analyses were conducted to assess the robustness and reliability of the model’s outcomes under varying parameters and hypothetical situations.

**Results:**

Annual LCS with volume-based LDCT detected 17,104 more lung cancer patients at early-stage among 207,885 screening population, leading to 8,761 premature lung cancer deaths averted. In addition, in contrast to no screening, LCS yielded 86,207 LYs gained and 50,207 incremental QALYs at an additional cost of €278,971,940, resulting in an ICER of €3,236 per LY and €5,505 per QALY, over a lifetime horizon. These estimates were robust in sensitivity analyses.

**Conclusions:**

LCS with volume-based LDCT, targeting an asymptomatic high-risk population, is highly cost-effective in Greece. Implementing LCS ensures efficient allocation of public healthcare resources while delivering substantial clinical benefits to lung cancer patients.

## Introduction

Lung cancer (LC) was the most common cancer diagnosed and the main cause of cancer mortality in 2020, accounting for around one in ten (11.4%) cancer diagnoses and one in five (18%) deaths, with an expected 2.2 million new cancer cases and 1.8 million deaths globally [1]. Cigarette smoking is the predominant risk factor for LC, and Greece has one of the highest smoking rates among European Union (EU) nations [2]. Consequently, 8,960 individuals were newly diagnosed with LC, leading to 7,662 deaths in a population of 10.4 million inhabitants in Greece in 2020 [3]. Therefore, LC is the foremost contributor to cancer-related mortality among male individuals in the Greek population, and it has the secondary position among their female counterparts after breast cancer [4]. Moreover, Greece has incurred approximately €38.2 million in hospital expenditure annually for treatment and management of conditions directly related to smoking [5]. Hence, there is an urgent need to address the substantial burden of disease and economic pressure imposed by LC.

Poor LC survival primarily results from late diagnosis, with a predominant proportion (56.9%) diagnosed at stage IV, in stark contrast to only 2.1% of cases detected at stage I, which is associated with a significantly better prospect with a 5-year survival rate ranging from 80% to 90% [6]. Research showed that lung cancer screening (LCS) is effective in detecting LC earlier and reducing LC-related mortality. The National Lung Screening Trial (NLST) and the Dutch-Belgian Lung Cancer Screening Study (Dutch acronym is NELSON) demonstrated that LCS with low-dose computed tomography (LDCT) resulted in a majority of LC cases being detected at stage I (58.3–64.9%), and only 6.8%–15.4% at stage IV [7,8]. Notably, these two studies reported that LCS led to an LC-related mortality reduction up to 20% and 26%, respectively [7,8]. Furthermore, the NELSON study implemented a nodule management protocol centred on the assessment of nodule volume growth, as opposed to relying on nodule diameter, resulting in less false-positive diagnoses when compared to the NLST [9,10].

There is no comprehensive nationwide LCS program in Greece yet, and consequently, studies assessing the cost-effectiveness of LCS are notably lacking in the Greek context. This study aims to investigate the clinical impact and economic viability associated with the adoption of LCS with volume-based LDCT in Greece, using insights from the NELSON study. Our intention is to clarify and disseminate information to policymakers, thereby promoting awareness and facilitating informed decision-making on the integration of LCS initiatives.

## Methods

A cost-effectiveness analysis (CEA) was performed using a model adapted from the core model previously developed by our research team. The structure and components of this model, as well as its underlying methodologies, have been thoroughly detailed in our prior publication [11]. Therefore, the subsequent sections focus on the various input variables used in this analysis, as the detailed model specifications and assumptions have been comprehensively described in prior work.

This study reported clinical outcomes, including the number of detected LC patients, LC-related deaths per stage, as well as premature LC-related deaths averted. Meanwhile, expenditure outcomes were also reported, such as costs for recruitment, screening, diagnostics, and treatment. Furthermore, the CEA used estimates of total expenditures, life years (LYs), and quality-adjusted life years (QALYs), to determine the incremental cost-effectiveness ratio (ICER), defined as costs per LY and costs per QALY.

### Model structure and design

A decision tree with an integrated Markov trace was developed to simulate the screening, identification, and treatment pathways for LC patients using Microsoft^®^ Excel^®^ (Version 2406 Build 16.0.17726.20078). This model was adapted from a previously validated framework designed to evaluate the cost-effectiveness of a national LCS program [11]. In our study, the model was tailored to reflect the context in Greece, incorporating local Greek data to ensure relevance and accuracy. The decision tree consisted of 2 arms, the screening arm and the no screening arm. In the screening arm, the eligible population was given the option to participate in LCS and received a volume-based LDCT. Negative scan results were followed by subsequent annually screenings. In the no screening arm, symptomatic LC patients were diagnosed through clinical presentation; notably, asymptomatic individuals with pre-clinical disease were considered as missed LC cases. LC patients in both arms, whether detected by LCS or identified based on symptoms, all entered the state-transition Markov model to simulate the long-term survival and costs. The Markov model consisted of three health states: a pre-progression state, a post-progression state, and a death state. Patients first entered the pre-progression state and could then either remain in this state or progress to the post-progression or death state. Eventually, all LC patients were absorbed in the death state. A three-month cycle length was chosen to reflect the typical treatment regimen for LC patients.

The base-case analysis was conducted from a payer’s perspective using a lifetime horizon. The cost-effectiveness analysis was conducted in accordance with the Professional Society for Health Economics and Outcomes Research (ISPOR) good practice report [12]. All costs were inflated to the year 2022 [13]. Both future health effect and costs were discounted at a rate of 3.5% annually, which is standard practice in Greece [14]. There is no standard willingness-to-pay (WTP) threshold in Greece, yet the most prominent practice has been one to three times growth domestic product (GDP) per capita [15]. GDP per capita in Greece was roughly €19,561 in 2022 [16]. In consultation with local experts, the WTP threshold was set to be €20,000 per QALY. Net Monetary Benefit (NMB) was calculated based on this WTP.

### Model inputs

The main model inputs are shown in Table 1. Greek local data was used to accurately reflect the Greek situation. International and alternative European data were considered only when local data was not available.

**Table 1:** Main model inputs for the base-case analysis.

Parameter		Base-case value	PSA distribution	Reference
**Discount rate for costs**		3.50%	Fixed	[14]
**Discount rate for health outcomes**		3.50%	Fixed	[14]
**Time horizon**		Lifetime (42 years)	Fixed	
**Screening effectiveness**				
**NELSON round 1**				
Regular scan	Negative	79.21%	Dirichlet	[17]
	Indeterminate	19.20%	Dirichlet	[17]
	Positive	1.59%	Dirichlet	[17]
Indeterminate scan	Negative	94.57%	Dirichlet	[17]
	Positive	5.43%	Dirichlet	[17]
True negative		99.93%	Dirichlet	[17]
False negative		0.07%	Dirichlet	[17]
True positive		38.67%	Dirichlet	[17]
False positive		61.33%	Dirichlet	[17]
Stage distribution				
Stage I		64.86%	Dirichlet	[18]
Stage II		9.46%	Dirichlet	[18]
Stage III		18.92%	Dirichlet	[18]
Stage IV		6.76%	Dirichlet	[18]
**NELSON round 2**				
Regular scan	Negative	92.17%	Dirichlet	[17]
	Indeterminate	6.58%	Dirichlet	[17]
	Positive	1.25%	Dirichlet	[17]
Indeterminate scan	Negative	91.23%	Dirichlet	[17]
	Positive	8.77%	Dirichlet	[17]
True negative		99.73%	Dirichlet	[17]
False negative		0.27%	Dirichlet	[17]
True positive*		44.35%	Dirichlet	[17]
False positive*		55.65%	Dirichlet	[17]
Stage distribution				
Stage I		75.86%	Dirichlet	[18]
Stage II		6.90%	Dirichlet	[18]
Stage III		13.79%	Dirichlet	[18]
Stage IV		3.45%	Dirichlet	[18]
**Mean age NELSON study**		58.00	Fixed	[17]
**Screening uptake rate**		30%	Beta	Expert opinions
**Epidemiology and demography**				
**Total population**		10,718,565	Gamma	[19]
Population aged 50–74 years		25.87%	Beta	[19]
Male		48.70%	Beta	[19]
Female		51.30%	Beta	[19]
Smoking rate		24.99%	Beta	[20]
Lung cancer incidence in patients aged 50–74 years		0.64%	Gamma	[21–23]
**Stage distribution for clinically presented patients**				
Stage I		9.59%	Dirichlet	[24]
Stage II		9.59%	Dirichlet	[24]
Stage III		28.14%	Dirichlet	[24]
Stage IV		52.67%	Dirichlet	[24]
**Survival**				
**Disease/progression-free survival (1-year disease/progression-free survival rate)**				
Stage I		87.80%	NA.	[25]
Stage II		81.79%	NA.	[25,26]
Stage III		48.92%	NA.	[27]
Stage IV		37.60%	NA.	[28–30]
**Overall survival (5-year survival rate)**				
Stage I		78.63%	NA.	[31]
Stage II		54.90%	NA.	[31]
Stage III		29.24%	NA.	[31]
Stage IV		5.91%	NA.	[31]
**Background mortality**				
Life expectancy by age		General population	Beta	[32]
**Utility**				
**Pre-progression state**				
Stage I		0.71	Beta	[33]
Stage II		0.68	Beta	[33]
Stage III		0.67	Beta	[33]
Stage IV		0.66	Beta	[33]
**Post-progression state**				
Stage I		0.67	Beta	[33]
Stage II		0.67	Beta	[33]
Stage III		0.66	Beta	[33]
Stage IV		0.66	Beta	[33]
**Lung cancer free participants** **				
Age-dependent utility values				
Aged 50–59 years		0.77	Beta	[34]
Aged 60–69 years		0.67	Beta	[34]
Aged 70–79 years		0.57	Beta	[34]
Aged 80 years onwards		0.52	Beta	[34]
**Costs**				
**Recruitment costs**				
Text message		€0.2	Gamma	Experts opinions
Consultation phone call		€4.0	Gamma	Experts opinions
**Screening costs**				
CT-scan costs (total)		€91	Gamma	[35]
CT-scan costs alone		€71	Gamma	[35]
Report reading costs		€20	Gamma	[35]
**Diagnostic costs per person**				
For screen detected patients		€651	Gamma	[36]
For clinically presented patients		€774	Gamma	[36]
**Treatment costs**				
First-line TC per 3 months (first year)				
Stage I		€2,458	Gamma	[24,35,37,38]
Stage II		€2,329	Gamma	[24,35,37,38]
Stage III		€7,920	Gamma	[24,35,37,38]
Stage IV		€8,959	Gamma	[24,35,37,38]
After-care TC for patients stay in the pre-progression state per 3 months (for the first 2 years)				
Stage I-IV		€100	Gamma	[35], experts opinions
After-care TC for patients stay in the pre-progression state per 3 months (after 2 years)				
Stage I-IV		€77	Gamma	[35], experts opinions
Second-line TC for patients who progressed (per patient)				
Stage I		€4,448	Gamma	[39,40]
Stage II		€4,448	Gamma	[39,40]
Stage III		€6,812	Gamma	[39,40]
Stage IV		€8,198	Gamma	[39,40]
After-care TC for patients who progressed per 3 months				
Stage I-IV		€423	Gamma	[39]
End of life costs (per patient)				
Stage I		€2,159	Gamma	[39]
Stage II		€2,159	Gamma	[39]
Stage III		€1,033	Gamma	[39]
Stage IV		€1,014	Gamma	[39]

NA, not applicable; PSA, probabilistic sensitivity analysis; TC, treatment costs.

*True positive refers to the proportion of true positive scans among the total of positive results, while false positive refers to the proportion of false positive scans among the total of positive results, the sum of them equals to 100.

**Lung cancer-free participants refer to people who either do not have lung cancer or have not been identified with lung cancer.

#### Simulated population.

The simulated population was defined as a high-risk group eligible for LCS, following the criteria outlined in the NELSON study, which included individuals aged 50–74 years and heavy smokers [8]. The population aged 50–74 years in Greece was estimated at approximately 2.77 million, and the smoking rate, determined by the number of daily smokers, was 24.99%, resulting in a number of 692,949 individuals eligible for LCS [20]. The screening population was then calculated based on a 30% uptake rate suggested by Greek clinical experts. Ultimately, 207,885 individuals were estimated to participate in the LCS [19].

#### Epidemiological inputs.

LC incidence in Greece was obtained from the World Health Organization (WHO) Cancer Today database [3]. The stage distribution at the time of diagnosis for clinically presented patients was derived from a retrospective study using data from a hospital-based registry of the Oncology Unit of ‘Sotiria’ Hospital in Athens, Greece. This cohort consisted of both non-small cell lung cancer (NSCLC) and small cell lung cancer (SCLC) patients, who died between 2015 and 2018 with a follow up of at least 6 months [24]. These epidemiological data were used to determine the transition probabilities in the decision tree for the no screening arm.

#### Screening effectiveness.

The screening effectiveness was based on the NELSON study outcomes, including screen-detected LC cases, false-positive cases, interval cancers, cancer-free participants, and LC stage distribution at diagnosis after screening detection, which showed that more than half of LC patients were detected at an early-stage (stage I-II) [17,18]. There are more patients diagnosed with LC in the screening arm. It is assumed that these exceeding LC cases were largely missed in the no screening arm. Therefore, to ensure a conservative estimate for the health benefits provide by LCS, these patients were also followed in the no screening arm, and defined as missed individuals. The missed individuals were assumed to have underlying early-stage LC disease.

#### Survival.

The time-dependent disease-free survival (DFS) for early-stage LC patients and the progression-free survival (PFS) for advanced-stage LC patients were used to determine the transition probabilities for LC patients moving from the pre-progression to the post-progression health state. DFS and PFS data were derived from both the real-world evidence [25] and multiple clinical trials [26–30].For stage I LC, DFS data was obtained from a retrospective study [25]; while for stage II, DFS data was synthesised from both this retrospective study and the clinical trial IMpower010, given that a predominant proportion of participants enrolled in this trial were stage II patients [25,26]. For stage III LC, PFS data was obtained from the PACIFIC trial [27]; whereas for stage IV patients, the PFS curve was estimated from several studies, including KEYNOTE-189, FLAURA, and Impower 133, reflecting different common treatments and LC types (Table A in S1 File) [28–30]. The International Association for the Study of Lung Cancer (IASLC) collected the LC overall survival (OS) data per stage at diagnosis from 16 countries, including Greece [31]. This OS data was used to determine the transition probability of LC patients from both the pre-progression and post-progression state to the death state. In addition, the missed individuals were assumed to follow the survival of stage II LC patients.

To reflect a lifetime horizon, survival extrapolation was performed based on the statistical method supported by the NICE Technical Support Document and Guyot et al. [41,42]. First, a simulated patient-level data set was derived, after which parametric extrapolation techniques were applied to predict long-term survival. Details on the distribution functions fitted per extrapolated survival curve are presented in Tables B and C in S1 File. In addition, the model took into account all-cause mortality based on the Greek life tables [32].

#### Utilities.

The health state utility values (HSUVs) were obtained from a study involving 10,000 lung cancer patients in the United States, representing a substantial sample within the population, as utility values for Greek LC patients were lacking (Table 1) [33].

For cancer-free participants, the Greek-specific age-dependent utility norm for general population was applied. These values were obtained from a study investigating 2,279 participants selected from the greater Athens area using a multistage stratified quota selection technique, with both EQ-5D-5L and EQ-5D-3L [34]. Furthermore, stage I and II LC patients who remained in the pre-progression state after 5 years of treatments and follow-up were considered clinically recovered. The utility norm was applied to these patients to correct for improved quality of life [43]. Moreover, as LC patients age, the utility norm may be lower than the constant LC-specific utilities after a certain age, indicating that aging has a greater impact on patients’ quality of life. In this case, a general population utility cap was used to ensure that LC utilities do not exceed the age-dependent population norms.

#### Costs.

For the screening arm, the total costs consisted of costs for recruitment, screening, diagnosis, and treatments. Based on the experts opinions, Greece is inclined to employ a population-based recruitment strategy for LCS, and it would entail a two-step process: a text message (SMS) was sent to the eligible population based on age (50–74 years), followed by a consultation phone call to assess eligibility of responsive individuals based on their smoking behaviour. The costs per text message and phone call were multiplied by the number of individuals contacted based on age and smoking status. This computation yielded the overall recruitment costs (Table 1). Screening costs were obtained from the National Tariff of Medical Practices published by the National Organization for Healthcare Services Provision (EOPYY) [35]. The diagnostic costs for clinically presented LC patients were derived from a study investigating a hospital-based registry in Greece from the year 2015 onwards, which included the costs for the clinical consultation, imaging examination, regular laboratory tests, and gene mutation tests [36]. For patients in the screening arm, the costs for the clinical consultation and CT scan were not included, as patients were asymptomatic and screen-detected [35] (Table 1).

The treatment costs were divided into different treatment phases. First-line treatment costs were synthesized based on a micro-costing approach. It included costs for systemic therapy, radiotherapy, surgery, post-surgery treatments, and hospitalization. For stage I and II, systemic therapy mainly involved chemotherapy; while for stage III and IV, it encompassed a combination of chemotherapy, immunotherapy, and targeted therapy with tyrosine kinase inhibitors (TKIs). Utilization per intervention was derived from a Greek hospital-based registry [24]. Unit costs for surgery and radiotherapy were obtained by EOPYY [35]. For chemotherapy, it was derived from a retrospective study measuring the initial treatment costs for LC patients [38]. For the immunotherapy and targeted therapy, unit costs per course were synthesized based on the treatment scheme recommended by the Guidelines of the Hellenic Society of Medical Oncologists (HESMO) [44], and the average weighted costs per medication were estimated based on the price per package [37] and the median treatment duration [45–49]. Details for this micro-costing synthesis can be found in Tables D and F in S1 File.

Costs for second-line treatment were obtained from a hospital-based retrospective study in Greece, which examined the costs incurred during the last six months before LC deaths [39]. These six-months costs, excluding the last month’s costs, were considered as the costs for the second-line treatments, as the median duration for the second-line treatment was approximately five months [40], and these costs were applied to patients who experienced disease progression in the model. Additionally, costs for the last subsequent month were used as the end-of-life costs and applied to all lung cancer deaths [39].

The after-care costs for patients without disease progression consisted of costs for chest CT scans, upper abdomen and brain CT scans [35]. In addition, patients received regular check-ups every six months for the first two years after the initial treatments, and every 12 months in the subsequent three years after the first two years. Meanwhile, for patients who progressed, the after-care costs consisted of the costs for the best supportive care, laboratory tests, imaging examination, and hospitalization [39]. All costs were inflated to the year 2022 [13] and are shown per phase in Table 1.

### Sensitivity analyses

One-way sensitivity analysis (OSA) was performed to explore which parameters were most influential on the ICER by individually varying the deterministic parameter values by 20%. In addition, a probabilistic sensitivity analysis (PSA) with 1,000 iterations was performed to examine the robustness of the analysis by varying all parameter values simultaneously, with results shown in a cost-effectiveness plane and a cost-effectiveness acceptability curve.

### Scenario analyses

Various scenarios were explored in the study. First, the impact of different LCS uptake rates was examined. Second, the mean age for the screening participants was varied from 55 to 74 years. Third, treatment costs were increased by 15%, as suggested by Greek clinical experts (details for the expert panellists can be found in Table F in S1 File), as LC patients received treatments from private healthcare facilities, where costs were higher than the public sector. Other scenarios explored the impact of the different screening rounds, shorter time horizons, and different discount rates for health effects and costs.

## Results

### Base-case results

A cohort of 207,885 high-risk Greek individuals was screened according to the volumetric protocol and NELSON study outcomes. Screening increased the number of early-stage (I-II) LC diagnoses by 17,104, while decreasing late-stage (III-IV) diagnoses by 8,884. When LCS was implemented, 8,761 premature LC deaths were expected to be averted (Table 2).

**Table 2:** Results from the base-case cost-effectiveness analysis.

	Screening		No screening		Incremental
**Clinical and health outcomes**					
**Lung cancer diagnoses**					
Total	73,528	(100%)	65,308	(100%)	8,220
Stage I	23,194	(32%)	6,266	(10%)	16,928
Stage II	6,443	(9%)	6,266	(10%)	176
Stage III	17,257	(23%)	18,381	(28%)	−1,124
Stage IV	26,634	(36%)	34,395	(53%)	−7,760
Missed Individuals	0	NA.	8,220	NA.	−8,220
Stage III and IV averted	8,884				
**Lung cancer deaths**					
Total	53,443		62,205		−8,761
Stage I	8,541		2,305		6,235
Stage II	3,391		3,296		95
Stage III	15,586		16,595		−1,009
Stage IV	25,926		33,479		−7,554
Missed individuals	0		6,529		−6,529
**Life years**					
Total	10,526,105		10,439,898		86,207
Stage I	185,081		49,822		135,259
Stage II	39,325		38,034		1,291
Stage III	46,895		49,810		−2,915
Stage IV	29,880		38,581		−8,702
Missed individuals	0		38,727		−38,727
Lung cancer free participants*	10,224,924		10,224,924		0
**Quality-adjusted life years**					
Total	6,701,652		6,650,973		50,679
Stage I	111,492		29,994		81,498
Stage II	23,758		22,958		800
Stage III	29,375		31,183		−1,808
Stage IV	18,977		24,505		−5,527
Missed individuals	0		24,284		−24,284
Lung cancer free participants*	6,518,050		6,518,050		0
**Costs**					
Total	€1,651,076,614		€1,372,104,674		€278,971,940
Recruitment costs	€1,386,117		NA.		€1,386,117
Screening costs	€230,407,174		NA.		€230,407,174
Diagnostic costs	€58,664,100		€39,621,566		€19,042,534
Treatment costs	€1,360,619,222		€1,332,483,108		€28,136,114
Stage I	€304,939,686		€82,179,083		€222,760,604
Stage II	€67,824,987		€65,728,492		€2,096,495
Stage III	€397,846,502		€422,740,766		€−24,894,265
Stage IV	€590,008,047		€761,834,767		€−171,826,720
**Health economic outcomes**					
ICER (costs per QALY)	€5,505				
NMB	€734,607,044				

NA, not applicable; ICER indicates incremental cost-effectiveness ratio; NMB, net monetary benefit.

*Lung cancer free participants refer to people who do not have lung cancer or have not been identified with lung cancer.

Over 17 annual screening rounds, the total recruitment, screening costs, and incremental diagnostic costs respectively amounted to €1,386,117 (€81,536 per annum), €230,407,174 (€13,553,363 per annum), and €19,042,534 (€1,120,149 per annum). Over a lifetime horizon, notable costs savings were observed in treatment expenses for patients diagnosed at stage III and IV, which amounted to €24,894,265 and €171,826,720, respectively (Table 2). Total incremental costs were €278,971,940, with 86,207 LYs and 50,679 QALYs gained, resulting in an ICER of €3,236 per LY and €5,505 per QALY. At a WTP threshold of €20,000 per QALY, the net monetary benefit (NMB) was €734,607,044 (Table 2).

### Sensitivity analyses

**Fig 1:**
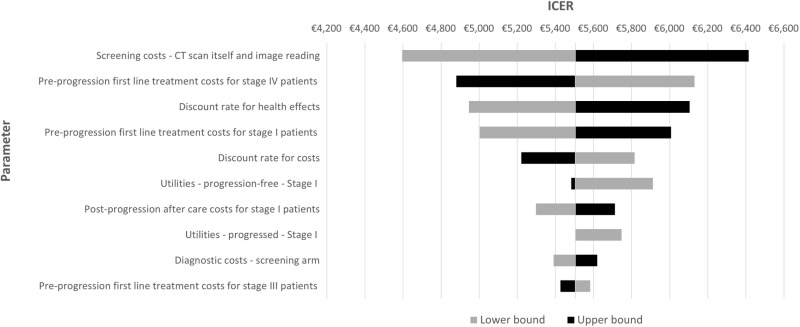
Tornado diagram from the one-way sensitivity analysis.

**Fig 2:**
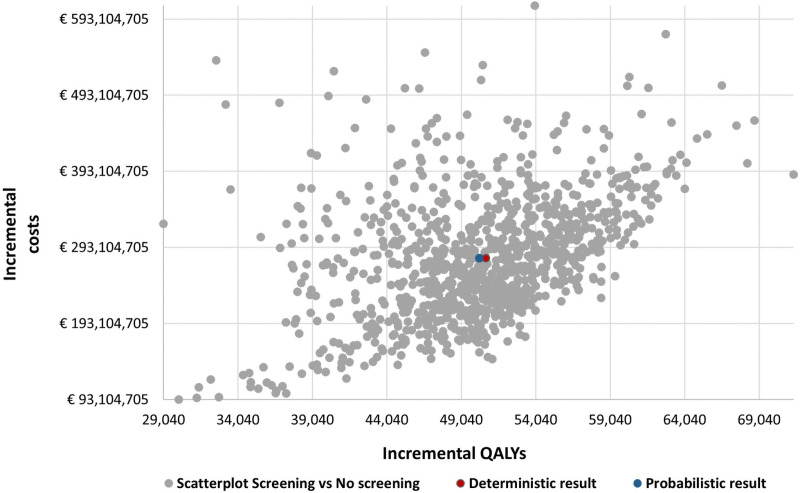
Incremental cost-effectiveness scatterplot from the probabilistic sensitivity analysis.

**Fig 3:**
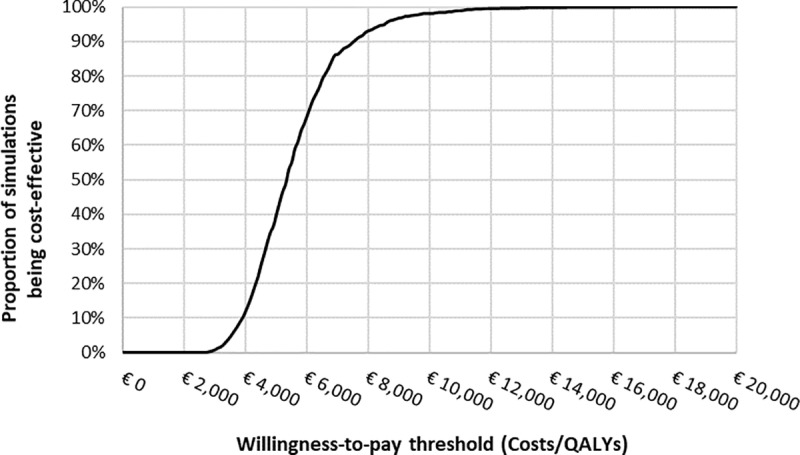
Cost-effectiveness acceptability curve from the probabilistic sensitivity analysis.

The OSA showed that the most influential input parameters affecting the ICER were the CT scan costs (CT costs itself and report reading costs), and the first-line treatment costs for stage I and IV patients (Fig 1). The PSA resulted in an average ICER of €5,551 per QALY with a 95% confidence interval of €3,316–€9,384 per QALY, indicating that most outcomes were below the WTP threshold and the analysis was robust (Fig 2). The cost-effectiveness acceptability curve demonstrated that the likelihood of LCS program being cost-effective increased as the WTP threshold escalated (Fig 3). The results of the scenario analyses are presented in Table 3. Varying the LCS uptake rate had a marginal impact on the ICER. Varying the mean age for the screening participants showed that screening a younger population (55 years) resulted in a lower ICER (€5,003 per QALY). All scenarios resulted in ICERs below the WTP threshold, except the scenario with a 5-year time horizon (€29,653 per QALY).

**Table 3:** Results from the scenario analyses.

Scenario	Screening		No screening		Incremental costs	Incremental QALYs	ICER
	Total costs	Total QALYs	Total costs	Total QALYs			
Base-case analysis	€1,651,076,614	6,701,652	€1,372,104,674	6,650,973	€278,971,940	50,679	€5,505
Screening uptake rate (25%)	€1,605,982,586	6,697,736	€1,373,413,540	6,655,504	€232,569,046	42,232	€5,507
Screening uptake rate (75%)	€2,056,922,864	6,736,895	€1,360,324,882	6,610,198	€696,597,982	126,697	€5,498
Mean age of the screening participants (55 years)	€1,687,920,464	7,392,357	€1,401,347,380	7,335,077	€286,573,083	57,280	€5,003
Mean age of the screening participants (60 years)	€1,621,707,943	6,206,877	€1,348,635,289	6,160,600	€273,072,654	46,277	€5,901
Mean age of the screening participants (65 years)	€1,532,497,222	5,237,495	€1,276,844,683	5,202,010	€255,652,539	35,484	€7,205
Mean age of the screening participants (70 years)	€1,401,359,231	4,149,059	€1,169,524,652	4,123,279	€231,834,578	25,779	€8,993
Time horizon (5 years)	€606,946,608	2,234,446	€509,819,381	2,231,170	€97,127,226	3,275	€29,653
Time horizon (10 years)	€1,100,281,663	3,867,873	€925,782,591	3,855,100	€174,499,072	12,774	€13,661
Time horizon (15 years)	€1,481,448,603	4,995,683	€1,243,931,568	4,971,210	€237,517,035	24,473	€9,705
Number of screening rounds (3 rounds)	€425,466,497	6,948,976	€343,873,115	6,933,448	€81,593,382	15,528	€5,255
Number of screening rounds (5 rounds)	€666,529,114	6,896,171	€545,360,871	6,872,229	€121,168,244	23,942	€5,061
Number of screening rounds (10 rounds)	€1,164,833,581	6,790,900	€962,845,832	6,751,671	€201,987,748	39,228	€5,149
Number of screening rounds (15 rounds)	€1,534,369,341	6,725,702	€1,273,652,308	6,677,435	€260,717,033	48,267	€5,402
Discount rates (cost: 0%, health outcome: 0%)	€2,155,700,237	9,797,378	€1,780,097,414	9,707,480	€375,602,824	89,898	€4,178
Discount rates (cost: 6%, health outcome: 6%)	€1,399,491,369	5,407,050	€1,166,381,556	5,371,469	€233,109,812	35,581	€6,552
Progressed stage IV patients incur a disutility of 0.1	€1,651,076,614	6,701,470	€1,372,104,674	6,650,737	€278,971,940	50,732	€5,499
Increase background mortality by 100%	€1,507,064,830	5,649,875	€1,255,431,156	5,612,095	€251,633,674	37,780	€6,661
LC incidence in population aged 50–74 years increase by 20% (0.88%)	€1,985,976,174	6,627,939	€1,856,553,173	6,568,571	€129,423,001	59,368	€2,180
LC incidence in population aged 50–74 years increase by 50% (1.02%)	€2,172,624,407	6,586,705	€2,128,691,114	6,530,585	€43,933,293	56,120	€783
LC incidence in population aged 50–74 years increase by 100% (1.35%)	€2,624,468,917	6,486,413	€2,794,259,895	6,445,323	€−169,790,978	41,090	€−4,132
Overall survival for missed individuals follows general population	€1,651,076,614	6,701,652	€1,372,104,674	6,680,352	€278,971,940	21,300	€13,097
Overall survival for missed individuals follows stage I patients	€1,651,076,614	6,701,652	€1,372,104,674	6,660,209	€278,971,940	41,443	€6,731
Overall survival for missed individuals follows stage III patients	€1,651,076,614	6,701,652	€1,372,104,674	6,641,145	€278,971,940	60,508	€4,611
Overall survival for missed individuals follows stage IV patients	€1,651,076,614	6,701,652	€1,372,104,674	6,632,850	€278,971,940	68,802	€4,055
AI-assisted imaging reading - report reading - £ 15	€1,638,432,164	6,701,652	€1,372,104,674	6,650,973	€266,327,489	50,679	€5,255
AI-assisted imaging reading - report reading - £ 10	€1,625,787,713	6,701,652	€1,372,104,674	6,650,973	€253,683,039	50,679	€5,006
AI-assisted imaging reading - report reading - £ 5	€1,613,143,263	6,701,652	€1,372,104,674	6,650,973	€241,038,589	50,679	€4,756
Doubled diagnostic costs for both arms	€1,709,740,714	6,701,652	€1,411,726,240	6,650,973	€298,014,474	50,679	€5,880
Increase the first-line treatments costs by 50%	€2,189,262,177	6,701,652	€1,934,984,660	6,650,973	€254,277,517	50,679	€5,017
Increase the second-line treatments costs by 50%	€1,686,853,663	6,701,652	€1,401,336,162	6,650,973	€285,517,501	50,679	€5,634
Increase both the first- and second-line treatments costs by 50%	€2,225,039,226	6,701,652	€1,964,216,148	6,650,973	€260,823,078	50,679	€5,147
Increase the first-line treatments costs for stage I LC patients by 50%	€1,651,076,614	6,701,652	€1,372,104,674	6,650,973	€278,971,940	50,679	€5,505
Increase the first-line treatments costs for stage IV LC patients by 50%	€1,605,982,586	6,697,736	€1,373,413,540	6,655,504	€232,569,046	42,232	€5,507
Increase the first-line treatments costs for stage III and IV LC patients by 50%	€2,056,922,864	6,736,895	€1,360,324,882	6,610,198	€696,597,982	126,697	€5,498

QALYs, quality-adjusted life years; ICER, incremental cost-effectiveness ratio; LC, lung cancer; AI, artificial intelligence; TC, treatment costs.

## Discussion

This study assessed the cost-effectiveness of volume-based low-dose CT screening versus no screening in Greece, resulting in an ICER of €5,505 per QALY from a healthcare payer perspective. Additionally, the results showed robustness in various uncertainty analyses. The cost-effectiveness of a targeted LCS in Greece has not been previously investigated, so our research provided a wealth of insights on this crucial topic for the first time. Additionally, our study showed that LCS facilitated early detection of LC, resulting in the prevention of 8,761 premature LC deaths in Greece. This fits well with the findings from a modelling study, which was also conducted in the Greek setting, and concluded that comprehensive LCS with LDCT resulted in fewer LC deaths and fewer life years lost, compared to the opportunistic screening [50]. However, the time horizon for the study was merely five years, and the authors reported solely the clinical outcomes – no quality of life and costs related to LCS were considered [50].

The one-way sensitivity analysis (OSA) showed that the unit costs per CT scan had the greatest impact on the cost-effectiveness of LCS, indicating that the key to implementing LCS in a more cost-effective framework was to have a proper budgetary restraint on the chest CT scans. New technologies with the potential to reduce this cost, or to achieve an economy of scale with current technology, are expected to further improve the cost-effectiveness of LCS. For example, artificial intelligence applications could support radiologists with reading and interpretation images, reducing time expenditure, and thereby lowering CT scan costs [51]. Other cost parameters, such as the first-line treatment costs for stage I and stage IV LC patients, the after-care costs for stage I LC patients who progressed, and diagnostic costs, also had a significant impact on the CE of LCS.

A study estimating the costs for Greek LC patients reported that diagnostic costs were €1,044 per patients, which was higher than the figures used in our model [52]. Therefore, we have employed a scenario analysis to double the diagnostic costs (€1,302 for the screening arm and €1,548 for the no screening arm), and this resulted in an ICER of €5,880 per QALY, indicating a marginal difference compared to the base-case ICER (€5,505 per QALY). Additionally, the same study reported that the total treatment costs per LC patients were €15,363, regardless of LC stage [52]. However, the study did not account for the novel treatments, like targeted drugs and immunotherapy, which were often associated with higher expenditure [52]. In our model, we included the costs for the targeted therapy and immunotherapy to reflect the current LC treatment paradigm. Furthermore, we provided more granularity in treatment costs; these were categorized in a longitudinal structure based on the chronological treatment phases (first-line treatment, second-line treatments, after-care for progression-free patients, after-care for progressed patients, and end-of-life treatments), which reflected the complete treatment pathway, and were synthesized per LC stage to illustrate the impact of LC stage shift (from late-stage to early-stage) achieved by LCS. Overall, estimating the cost of resources for LC patients is a very complex procedure, especially in the Greek context, as appropriate electronic recording of patient data is lacking in most hospitals. More research needs to be done in this field to ensure a more accurate budget planning and allocation for LCS.

For the base-case analysis, a screening rate of 30% was used based on consultation with Greek experts, who have expressed concerns about the uptake of LCS. Though the trial data have been favourable, the NELSON study had a participation rate of 51% [17], and the Lung Screen Uptake Trial (LSUT) performed in London, as a more recent example, showed a 53% participation rate [53]. However, low uptake of screening has been observed in the United Stated since its approval by the United States Preventive Services Task Force (USPSTF) in 2013 - only 2% of the eligible high-risk individuals were estimated to be screened in 2016 [54]. In our model, several scenario analyses were conducted to explore the impact of the LCS uptake on the cost-effectiveness outcomes. When the uptake rate increased from 25% to 75%, the additional QALYs gained per patient increased significantly from 0.58 to 1.49. However, varying the LCS uptake rate had a marginal impact on the ICER, as the total costs and QALYs changed proportionally according to the size of the screening population. In light of these findings, it becomes evident that increased uptake of LCS may yield greater benefits for LC patients. Accordingly, it is crucial to ensure comprehensive use of LCS in real-world applications that extend beyond the limits of controlled clinical trials.

There is no official recommendation from the Greek Ministry of Health for LCS with LDCT for high-risk individuals yet, despite evidence provided by two large LCS trials, NLST and NELSON, that showed a significant LC-related mortality reduction. There are concerns about budgetary constraints and cost-effectiveness associated with the implementation of a nationwide LCS program. Our research provides insights in this area. There is no standard WTP threshold to promote efficient use of healthcare resources in Greece. Most cost-effectiveness studies follow the WHO recommendation of a threshold of less than three times the national annual GDP per capita, and interventions costing less than one time the GDP per capita are deemed highly cost-effective. Therefore, the WTP was set to be €20,000 per QALY as a conservative estimate in the model, as GDP per capita in Greece was roughly €19,561 in 2022 [16]. A systematic review on WTP thresholds used in Greece reported a median value for oncological studies of €51,000 per QALY [15]. Although the base case ICER in our analysis is already considered highly cost-effective at a WTP threshold of €20,000, this is even more so considering an even higher threshold. Hence, LCS is deemed highly cost-effective in the Greek healthcare setting. Nonetheless, healthcare system structures and differences in resource allocation play a significant role in the cost-effectiveness of LCS programs. Systems with well-integrated screening infrastructure and efficient resource distribution may enhance cost-effectiveness by facilitating streamlined patient pathways and ensuring timely interventions. It is recommended that policymakers invest in robust screening infrastructure and equitable resource allocation to optimize cost-effectiveness.

The main strength of this study is its novelty - it is the first analysis to assess the cost-effectiveness of a nationwide targeted LCS in Greece, based on the up-to-date NELSON study results and local epidemiological and cost data. The limitations of the study include the absence of local health state utility values, which are essential for accurately assessing the quality of life for LC patients by stage, as well as the lack of Greek-specific OS data. However, the study used OS data reported by IASLC, which incorporates data from 16 European countries, including Greece [31]. Future research should focus on providing evidence on the quality of life and survival of LC patients in the local setting, which is pivotal for demonstrating the benefits attributed to LCS.

## Conclusions

Lung cancer screening with volume-based LDCT, targeting an asymptomatic high-risk population, is highly cost-effective in Greece, based on a WTP threshold of €20,000 per QALY. Implementation of LCS ensures efficient allocation of public healthcare resources while delivering substantial clinical benefits to LC patients.

## Supporting information

S1 File Tables with supporting information. (PDF)

S2 File Values used to build graphs. (XLSX)

S3 File The points extracted from images for overall survival. (XLSX)

S4 File The points extracted from images for PFS. (XLSX)
