# Supplementary material for: Lung cancer screening with volume computed tomography is cost-effective in Greece
Source: PLoS One. 2025 Mar 5;20(3):e0316351. doi: 10.1371/journal.pone.0316351 (PMC11882067; doi:10.1371/journal.pone.0316351)
Supplement: S1 File — (PDF) [file pone.0316351.s001.pdf]

Table A. Clinical trials used to synthesise the progression-free survival data for stage IV lung cancer patients.

Trial	Patient enrolled	Intervention	Trial design	Weight*
KEYNOTE-189 (28)	616 non-actionable mutations NSCLC patients (advanced stage)	Pembrolizumab + chemotherapy	Phase III double-blind RCT	68%
FLAURA (30)	279 EGFR-mutated NSCLC patients (advanced stage)	Osimertinib	Phase III double-blind RCT	17%
IMpower133 (29)	403 SCLC patients (advanced stage)	Atezolizumab + chemotherapy	Phase III double-blind RCT	15%
*NSCLC, non-small cell lung cancer; RCT, randomised control trial; SCLC, small cell lung cancer. * the weights used to synthesize the survival curves from various clinical trials were based on the epidemiology of lung cancer patients and the prevalence of the gene mutation.*				

Table B. The fitted distribution functions per overall survival curve.

**Stage I**	**Fitted distribution**
Stage IA1	Log-Logistic
Stage IA2	Weibull
Stage IA3	Weibull
Stage IB	Weibull
**Stage II**	
Stage IIA	Weibull
Stage IIB	Weibull
**Stage III**	
Stage IIIA	Weibull
Stage IIIB	Weibull
Stage IIIC	Log-Logistic
**Stage IV**	
Stage IVA	Weibull
Stage IVB	Weibull

Table C. The fitted distribution functions per disease/progression-free survival curve

	**Explanation for curves**	**Fitted distribution**
**Stage I**		
Curve A	Stage I-II (DFS) upstaged	Log-Logistic
Curve B	Stage I-II (DFS) non-upstaged	Log-Logistic
**Stage II**		
Curve A'	Stage I-II (DFS) upstaged	Log-Logistic
Curve B'	Stage I-II (DFS) non-upstaged	Log-Logistic
Curve C'	stage II (DFS) - IMpower010 - best supportive care arm	Log-Normal
**Stage III**		
Curve A"	Stage III (PFS) PACIFIC - Durvalumab arm	Log-Normal
Curve B"	Stage III (PFS) PACIFIC - Control arm	Log-Normal
**Stage IV**		

Curve A'''	Stage IV (PFS) non-EGFR mutated NSCLC (Gandhi et al.)	Log-Logistic
Curve B'''	Stage IV (PFS) EGFR mutated NSCLC (Soria et al.)	Log-Normal
Curve C'''	Stage IV (PFS) SCLC (Horn et al.)	Log-Logistic

Table D. The micro-costing approach to synthesize the first-line treatment costs per lung cancer stage.

Item	Utilization	Unit costs (per year, 2022)	Source
**Stage I**	100%	€9,834	
**Systemic therapy**[*]	2%	€781	Ibarrondo et al., 2022
**Radiotherapy**[*]	0%	€6,500	EOPYY, 2022
**Surgery with or without neoadjuvant**[*]	98%	€8,000	EOPYY, 2022
**First post-surgery treatment**	100%	€1,017	
**No post-surgery treatment**	36%	€-	
**Systemic therapy**	11%	€781	Ibarrondo et al., 2022
**Radiotherapy**	9%	€6,500	EOPYY, 2022
**Adjuvant therapy**	43%	€781	Ibarrondo et al., 2022
**Hospitalization**[*]	100%	€1,000	Expert opinions
**Stage II**	100%	€9,316	
**Systemic therapy**[*]	5%	€7,22	Ibarrondo et al., 2022
**Radiotherapy**[*]	0%	€6,500	EOPYY, 2022
**Surgery with or without neoadjuvant**[*]	95%	€8,000	EOPYY, 2022
**First post-surgery treatment**	100%	€687	
**No post-surgery treatment**	5%	€-	
**Systemic therapy**	7%	€7,22	Ibarrondo et al., 2022
**Radiotherapy**	0%	€6,500	EOPYY, 2022
**Adjuvant therapy**	88%	€722	Ibarrondo et al., 2022
**Hospitalization**[*]	100%	€1,000	Expert opinions
**Stage III**	100%	€13,802	
**Systemic therapy**[*+]	68%	€13,918	
**Chemotherapy**	20%	€1,021	Ibarrondo et al., 2022
**Chemo+immunotherapy**	50%	€50,661	Galinos, 2022
**Immunotherapy**	20%	€50,010	Galinos, 2022
**Targeted therapy with TKIs**	10%	€31,366	Galinos, 2022
**Radiotherapy**[*]	5%	€6,500	EOPYY, 2022
**Surgery with or without neoadjuvant**[*]	27%	€8,000	EOPYY, 2022
**First post-surgery treatment**	100%	€3,090	
**No post-surgery treatment**	15%	€-	
**Systemic therapy**	15%	€13,918	Ibarrondo et al., 2022; Galinos, 2022
**Radiotherapy**	6%	€6,500	EOPYY, 2022

**Adjuvant therapy**	65%	€1,021	Ibarrondo et al., 2022
**Hospitalization***	100%	€1,000	Expert opinions
**Stage IV**	100%	€15,403	
**Systemic therapy*+**	85%	€15,453	
**Chemotherapy**	20%	€3,213	Ibarrondo et al., 2022
**Chemo+immunotherapy**	50%	€50,661	Galinos, 2022
**Immunotherapy**	20%	€50,010	Galinos, 2022
**Targeted therapy with TKIs**	10%	€31,366	Galinos, 2022
**Radiotherapy***	12%	€6,500	EOPYY, 2022
**Surgery with or without neoadjuvant***	2%	€8,000	EOPYY, 2022
**First post-surgery treatment**	100%	€9,893	
**No post-surgery treatment**	17%	€-	
**Systemic therapy**	50%	€15,453	Ibarrondo et al., 2022; Galinos, 2022
**Radiotherapy**	33%	€6,500	EOPYY, 2022
**Adjuvant therapy**	0%	€3,213	Ibarrondo et al., 2022
**Hospitalization***	100%	€1,000	Expert opinions

*Items marked with * are the initial treatment received in parallel after diagnosis.
+ the utilization for chemotherapy, chemo+immunotherapy, immunotherapy, and targeting therapy for stage III and IV patients is based on the expert opinions.
Systemic therapy primarily involved chemotherapy for stage I and II, whereas for stage III and IV, it encompassed a combination of chemotherapy, immunotherapy, and targeted therapy with tyrosine kinase inhibitors (TKIs).

Table E. Costs estimation for the immunotherapy and targeting therapy.

Drug	Unit costs	Cost from a payer perspective *	Cost per 30 days	Packaging	Recommended dose	Average treatment duration (months)	Source
**Immunotherapy**							
Pembrolizumab (KEYTRUDA®) - monotherapy	€2,332	€2,216	€6,330	100mg/4ml	200 mg every 3 weeks	7.9	KEYNOTE-024
Pembrolizumab (KEYTRUDA®) + Chemotherapy	€2,332	€2,216	€6,330	100mg/4ml	200 mg every 3 weeks	9.8	KEYNOTE-189
Atezolizumab (Tecentriq) + Chemotherapy	€3,063	€2,910	€6,236	1200mg/20ml vial	1200 mg every 3 weeks	6.3	IMpower150
**Tyrosine kinase inhibitors**							
Erlotinib (TARCEVA®)	€1,324	€1,258	€1,258	150mg/tab - 30 tablets	150mg per day	13.5	Stinchcombe et al., 2019

Gefitinib (IRESSA®)	€1,351	€1,284	€1,284	250mg/tab - 30 tablets	250mg per day	11.5	FLAURA Soria et al.
Afatinib (GIOTRIF®)	€1,436	€1,364	€1,462	40mg/tab - 30 tablets	40mg per day	11.0	LUX-Lung 7
Osimertinib (TAGRISSO®)	€4,705	€4,470	€4,470	80mg/tab - 30 tablets	80mg per day	16.2	MOK: ITT population
Crizotinib (XALKORI®)	€3,535	€3,358	€3,358	250mg/cap - 60 tablets	500mg per day	10.9	PROFILE 104

* To reflect the cost from a payer perspective, a discount of 5% was applied to the original unit costs per meditation to derive the hospital price, according to the Official Government Gazette, Law 3918/2017 (59).

Table F. Details for the Greek expert's panel.

Name	**Affiliate**
**Prof. C.N. Foroulis**	Aristotle University of Thessaloniki, A.H.E.P.A. University Hospital
**Prof. K.N. Syrigos**	Athens University School of Medicine
**Prof. Sofia Agelaki**	University of Crete
**Dr. I.Gkiozos**	Sotiria Thoracic Diseases Hospital of Athens
**Dr. E. Zervas**	Sotiria Thoracic Diseases Hospital of Athens

**Dr. S. Tavernaraki**	Sotiria Thoracic Diseases Hospital of Athens
**MD. PhD. S. Lampaki**	Aristotle University of Thessaloniki
**Dr. G. Kourlaba**	University of Peloponnese
